# Gustatory and olfactory dysfunction in laryngectomized patients

**DOI:** 10.5935/1808-8694.20130099

**Published:** 2015-10-08

**Authors:** Ada Salvetti Cavalcanti Caldas, Vera Lúcia Dutra Facundes, Daniele Andrade da Cunha, Patrícia Maria Mendes Balata, Leila Bastos Leal, Hilton Justino da Silva

**Affiliations:** a MSc. - Occupational Therapist.; b PhD in Neuropsychiatry and Behavioral sciences; Associate Professor - Department of Occupational Therapy - Federal University of Pernambuco.; c PhD in nutrition; Substitute Professor - Department of Speech Pathology - Federal University of Pernambuco.; d PhD in Neuropsychiatry and Behavioral sciences; Speech and Hearing Therapist.; e PhD in Pharmaceutical Sciences; Associate Professor - Department of Pharmacy - Federal University of Pernambuco.; f PhD in Nutrition; Associate Professor - Department of Speech Therapy - Federal University of Pernambuco. MSc. in Pathology - Federal University of Pernambuco - UFPE - Recife (PE) - Brazil.

**Keywords:** laryngectomy, olfaction disorders, taste disorders

## Abstract

After total laryngectomy surgery, nasal airflow is moved permanently to the tracheostomy opening, compromising the contact of odorant molecules with the nasal cavity, which may reflect changes in the olfactory and gustatory perception in these individuals.

**Objective:**

To evaluate the functions of smell and taste in total laryngectomized patients. Study design: a study of series.

**Method:**

The sample included a group of 25 patients submitted to total laryngectomy and another group of 25 patients who did not underwent the procedure. The taste function was evaluated by gustatory strips of filter paper. To assess the olfactory function we employed the Brief Smell Identification Test.

**Results:**

Among the laryngectomized patients there was more hypogeusia (80%, *p* < 0.05), as well as hyposmia (88%, *p* < 0.001), alone and concomitant (72%, *p* < 0.001). Concerning flavor discrimination, the bitter taste did not differ between the groups - which was different from the other flavors. In the olfactory aspect, laryngectomized patients performed worse in detecting warning and food-related odors. We found that a history of smoking and alcohol consumption were significantly more frequent among laryngectomized patients.

**Conclusion:**

We found a decrease of gustatory and olfactory functions in total laryngectomized patients in this study.

## INTRODUCTION

After total laryngectomy surgery, nasal airflow is permanently transferred to the tracheostome, compromising the arrival of odorant molecules to the nasal cavity^1^, ^2^. The decrease in olfactory (hyposmia) and gustatory (hypogeusia) perceptions of individuals undergoing this intervention is often reported in the literature^3^, ^4^. Currently, it is considered that the laryngectomy may cause these changes due to the interruption that occurs in the respiratory tract, as well as by changes in the epithelial structure of the nasal mucosa and in the sensorineural feedback^5^, ^6^.

These sensory changes are less frequently investigated in clinical practice, since the loss of verbal communication, pulmonary complications and the psychosocial problems are evident after this surgery, and more often rehabilitated.

The present study aimed to evaluate the smell and taste perceptions in patients submitted to total laryngectomy, compared with non-laryngectomized individuals, by means of two quantitative tests.

## METHOD

### Study Group

We had a group of 25 patients who underwent total laryngectomy for cancer and a comparison group of 25 individuals without laryngectomy, regardless of gender and education.

The exclusion criteria for both groups were: a history of smell and taste disorders, use of medications that could impair the functions analyzed, as well as if at the time of collection the patient had rhinitis, sinusitis, and inflammatory processes in the stomatognathic system.

We had an odds-ratio of 10.22 for the group of laryngectomized individuals, our sample counted with 50 subjects, representing a 99.9% proof power, with a significance level of 0.05.

### Smell Test

To assess the olfactory function we used The Brief Smell Identification Test - B-SIT (Sensonics Inc.^®^, Haddon Hts., NJ 08035) from the University of Pensilvânia^7^. The test consists of presenting 12 scents (cinnamon, turpentine, lemon, smoke, chocolate, roses, paint thinner, banana, pineapple, gasoline, soap, onions), contained in microcapsules of urea-formaldehyde polymers of 10-50 micrometers, fixed in strips contained in the bottom corner of 12 pages of a single booklet.

The test was of rapid administration, establishing a relative degree of olfactory function loss through percentiles.

### Taste test

The instrument used to evaluate the gustatory function was based on the test validated by Muller *et al*.^8^. Strips of filter paper 8 cm long and 2 cm^2^ were impregnated with different concentrations of the following flavors: salty, sweet, bitter, sour; there were also two strips with distilled water (unflavored) used to validate the study; totaling 18 strips. We used the following concentrations: sour - 0.3 g/ml, 0.165 g/ml, 0.09 g/ml and 0.05 g/ml citric acid; bitter - 0.006 g/ml 0.0024 g/ml, 0.0009 g/mL to 0.0004 g/ml quinine sulphate; sweet - 0.4 g/ml, 0.2 g/ml, 0.1 g/ml and 0.05 g/ml sucrose; salty - 0.25 g/ml 0.1 g/ml 0.04 g/ml and 0.016 g/ml sodium chloride.

The strips were placed on the middle of the volunteer's tongue at a distance of approximately 1.5 cm from the tip of the tongue, and the test began with the lowest concentration. After evaluating each strip, the volunteer rinsed his mouth with water to remove any residue.

In accordance with recommendations in the literature,^8^ the taste test was conducted at least one hour after the last feeding, ingestion of any drink (except for water), having smoked or having brushed the teeth.

### Statistical Analysis

The data was organized in an Excel^®^ spreadsheet and analyzed using the SPSS version 17.0 software. For data analysis purposes we used the chi-square, Fisher's exact and ANOVA tests.

To classify the study subjects from both groups, as for their gustatory function, we used nine correct answers out of a total of 16 concentrations tested as the cutting point; classifying as hypogeusia a total less than or equal to 9; and normogeusia a total number of correct answers greater than 9. For sweet, salty and sour gustatory stimuli, the perception was classified as hypogeusia when the total number of correct answers was less than or equal to two. For the bitter gustatory stimulus, hypogeusia was considered when there was one or less correct answer^8^.

Proper olfactory function, according to age and gender, was classified as hyposmia when the total number of correct answers vis-à-vis the olfactory stimuli was less than nine, following the guidelines for B-SIT^®^ application^7^.

Variables related to age and number of correct answers vis-à-vis the olfactory and gustatory stimuli were expressed as a mean, standard error of the mean; with respective confidence intervals at 95% level.

To compare the mean values between the groups, we employed the ANOVA test and, to compare the distributions of absolute and relative frequencies, we used the Person's chi square or Fisher's exact tests. We employed a significance level of 0.05 for all tests.

This study was approved by the Ethics in Human Research Committee, #33/2010 (CAAE: 0015.0.447.000-10) and all participants signed an Informed Consent Form (ICF).

## RESULTS

Most of the laryngectomized volunteers had incomplete elementary education (21, 84.0%) (*p* < 0.001), a more frequent history of smoking (84%) and alcohol intake (60%) (*p* < 0.001) and larger number of missing teeth (*p* < 0.05) (Table 1).

**Table 1:** Distribution of comparisons between the laryngectomized patients' group and the comparison group by sampling describing variables.

Variables	Laryngectomized (n) (%)	Comparison (n) (%)	p-value
Gender			0.004^1^

Males	20 (80.0)	10 (40.0)	
Females	05 (20.0)	15 (60.0)	

Age range			0.128^2^

45-49	02 (10.0)	01 (04.0)	
50-54	05 (25.0)	12 (48.0)	
55-59	05 (20.0)	06 (24.0)	
60-64	04 (10.0)	05 (20.0)	
65-69	07 (25.0)	01 (4.0)	
70-74	01 (5.0)	-	
> 74	01 (5.0)	-	

Education			< 0.001^1^

Illiterate	07 (28.0)	-	
Incomplete elementary	14 (56.0)	01 (4.0)	
Complete elementary	02 (8.0)	-	
Complete high school	02 (8.0)	08 (32.0)	
Incomplete higher	-	02 (8.00)	
Complete higher	-	05 (20.0)	
Graduate	-	09 (36.0)	

Smoking			< 0.001^1^

Former smoker	21 (84.0)	09 (36.0)	
Never smoked	04 (16.0)	15 (60.0)	
Smoker	-	01 (4.00)	

Alcohol consumption			< 0.001^3^

Former alcoholic drinker	15 (60.0)	02 (8.00)	
Non-alcoholic	10 (40.0)	23 (92.0)	

Edentulous			0.480^3^

No	04 (16.0)	06 (24.0)	
Yes	21 (84.0)	19 (76.0)	

Total edentulous	12 (48.0)	18 (72.0)	0.007^4^
Partially edentulous	09 (36.0)	01 (4.00)	

1 p-values calculated with the Chi Square test;

2 p-values were calculated using the Fisher's exact test;

3 Comparison as to the presence or absence of teeth, by Fisher's exact test;

4 Comparison of the type of dental prosthesis, by the Fisher's exact test.

In gustatory perception tests, the mean score of correct answers among the laryngectomized patients equaled 7.2 ± 0.48 points, ranging between 1-11 points, while in the comparison group, the mean was 10.5 ± 0.7 points, ranging from 4 to 16 points. This difference was significant (F = 15.695, *p* < 0.001).

Considering the cutoff point for gustatory capacity adjustment^8^, we noticed that the laryngectomized group had significantly more frequent hypogeusia (20; 80.0%) than the comparison group (9; 36.0%) (c^2^ = 5. 88, *p* = 0.015) (Table 2).

**Table 2:** Distribution of comparisons between the laryngectomized patients' group and the comparison group according to gustatory and olfactory discrimination by gender, educational level, smoking and alcohol consumption.

Variables	Condition	Laryngectomized (n) (%)	Comparison (n) (%)	p-value
	Hypogeusia	20 (80.0)	9 (36.0)	0.015^2^
	Gender			0.108^1^
	Males	17 (68.0)	5 (20.0)	
	Females	3 (12.0)	4 (16.0)	
	Education			< 0.001^1^
	Up to high school	20 (80.0)	3 (12.0)	
Gustatory perception	Higher education	-	6 (24.0)	
	Smoking			0.001^1^
	Yes	18 (72.0)	2 (8.0)	
	No	2 (8.0)	7 (28.0)	
	Alcohol drinking			0.009^1^
	Former drinker	13 (52.0)	1 (4.0)	
	Not an alcoholic drinker	7 (28.0)	8 (32.0)	

	Hyposmia	22 (88.0)	10 (40.0)	< 0.001^2^
	Gender			0.007^1^
	Males	18 (72.0)	3 (12.0)	
	Females	4 (16.0)	7 (28.0)	
	Education			< 0.001^1^
	Up to high school	22 (88.0)	4 (16.0)	
Olfactory perception	Higher education	-	6 (24.0)	
	Smoking			0.027^1^
	Yes	18 (72.0)	4 (16.0)	
	No	4 (16,0)	6 (24.0)	
	Alcoholic drinker			0.001^1^
	Former drinker	13 (52.0)	-	
	Not an alcoholic drinker	9 (36.0)	10 (40.0)	

1 *p* values calculated with the Fisher's exact test;

2 p-values calculated with the Chi Square test.

Hypogeusia was more frequent among laryngectomized with schooling up to middle level and a history of smoking and alcohol consumption, when compared with non-laryngectomized individuals and not all of these variables were statistically significant (Table 2).

Among the sweet, salty and sour tastes, the laryngectomized group had the most frequent percentage of identification errors, while for the bitter taste it was equal for both groups (Table 3).

**Table 3:** Distribution of comparisons between the group of laryngectomized patients and the comparison group according to gustatory perception.

Flavors	Substances and concentrations	Laryngectomized (n) (%)	Comparison (n) (%)	p-value
	Sucrose 0.05g/mL			0.695
	Wrong Answer	23 (92.0)	23 (92.0)	
	Correct Answer	2 (8.0)	2 (8.0)	
	Sucrose 0.1g/mL			0.040
	Wrong Answer	13 (52.0)	6 (24.0)	
Sweet	Correct Answer	12 (48.0)	19 (76.0)	
	Sucrose 0.2g/mL			0.025
	Wrong Answer	10 (40.0)	3 (12.0)	
	Correct Answer	15 (60.0)	22 (88.0)	
	Sucrose 0.4g/mL			0.334
	Wrong Answer	4 (16.0)	2 (8.0)	
	Correct Answer	21 (84.0)	23 (92.0)	

	Sodium Chloride 0.016 g/mL			< 0.001
	Wrong Answer	19 (76.0)	6 (24.0)	
	Correct Answer	6 (24.0)	19 (76.0)	
	Sodium Chloride 0.04 g/mL			0.011
	Wrong Answer	18 (72.0)	9 (36.0)	
Salty	Correct Answer	7 (28.0)	16 (64.0)	
	Sodium Chloride 0.1 g/mL			0.040
	Wrong Answer	13 (52.0)	6 (24.0)	
	Correct Answer	12 (48.0)	19 (76.0)	
	Sodium Chloride 0.25 g/mL			0.064
	Wrong Answer	11 (44.0)	5 (20.0)	
	Correct Answer	14 (56.0)	20 (80.0)	
	Citric Acid 0.0125 g/mL			0.005
	Wrong Answer	18 (72.0)	8 (32.0)	
	Correct Answer	7 (28.0)	17 (68.0)	
	Citric Acid 0.0225 g/mL			0.070
	Wrong Answer	12 (48.0)	6 (24.0)	
Sour	Correct Answer	13 (52.0)	19 (76.0)	
	Citric Acid 0.041 g/mL			0.122
	Wrong Answer	12 (48.0)	7 (28.0)	
	Correct Answer	13 (52.0)	18 (72.0)	
	Citric Acid 0.075 g/mL			0.189
	Wrong Answer	11(44.0)	7 (28.0)	
	Correct Answer	14 (56.0)	18 (72.0)	

	Quinine sulphate 0.0001 g/mL			0.500
	Wrong Answer	21 (84.0)	20 (80.0)	
Bitter	Correct Answer	4 (16.0)	5 (20.0)	
	Quinine sulphate 0.0002 g/mL			0.182
	Wrong Answer	19 (76.0)	15 (60.0)	
	Correct Answer	6 (24.0)	10 (40.0)	
	Quinine sulphate 0.0006 g/mL			0.381
	Wrong Answer	7 (28.0)	9 (36.0)	
	Correct Answer	18 (72.0)	16 (64.0)	
	Quinine sulphate 0.0015 g/mL			0.173
	Wrong Answer	9 (36.0)	5 (20.0)	
	Correct Answer	16 (64.0)	20 (80.0)	

The *p* values were calculated using the Fisher's exact test.

In the olfactory discrimination test, the laryngectomized group had a mean level of correct answers equal to 6.0 ± 0.5, points, ranging between 3-11 points, differing significantly from the comparison group, which obtained a mean level of correct answers equal to 9.1 ± 0.3 points, ranging from 6 to 11 points (F = 31.937, *p* < 0.001).

Similarly to that observed on the gustatory assessment, we found that hyposmia was more frequent among males with mid-level education, as well as in the presence of a history of smoking and alcohol consumption among laryngectomized subjects and all these differences were significant (Table 2).

Considering nine points as the cutoff point for olfactory capacity adaptation, according to gender and age^7^, we noticed that 15 (60.0%) individuals from the comparison group had normal olfactory perception for their age and gender, compared with three (12%) from the laryngectomy group. In the comparison group, 10 (40%) subjects had abnormal olfactory discrimination for age and gender - a percentage significantly lower than the 88% in the laryngectomized group (c^²^ = 12.50; *p* < 0.001) (Table 2).

Table 4 shows the breakdown of this difference. Laryngectomized patients more often did not identify odors of smoke, chocolate, rose, turpentine, banana, pineapple, onions and gasoline, and all these differences were significant. Although the laryngectomized have presented lower scores in turpentine and lemon odor identification, differences in relation to the comparison group were not significant.

**Table 4:** Distribution of comparisons between the group of laryngectomized patients and the comparison group according to the olfactory performance.

Olfactory perception	Laryngectomized n (%)	Comparison n (%)	*p*-value
Cinnamon			< 0.001

Wrong answer	6 (24.0)	22 (88.0)	
Correct answer	19 (76.0)	3 (12.0)	

Turpentine			0.085

Wrong answer	22 (88.0)	17 (68.0)	
Correct answer	3 (12.0)	8 (32.0)	

Lemon			0.078

Wrong answer	15 (60.0)	9 (36.0)	
Correct answer	10 (40.0)	16 (64.0)	

Smoke			0.002

Wrong answer	10 (40.0)	1 (4.0)	
Correct answer	15 (60.0)	24 (96.0)	

Chocolate			0.001

Wrong answer	13 (52.0)	2 (8.0)	
Correct answer	12 (48.0)	23 (92.0)	

Roses			< 0.001

Wrong answer	18 (72.0)	2 (8.0)	
Correct answer	7 (28.0)	23 (92.0)	

Paint thinner			< 0.001

Wrong answer	17 (68.0)	4 (16.0)	
Correct answer	8 (32.0)	21 (84.0)	

Banana			0.003

Wrong answer	13 (52.0)	3 (12.0)	
Correct answer	12 (48.0)	22 (88.0)	

Pineapple			< 0.001

Wrong answer	10 (40.0)	-	
Correct answer	15 (60.0)	25 (100.0)	

Gasoline			0.005

Wrong answer	9 (36.0)	1 (4.0)	
Correct answer	16 (64.0)	24 (96.0)	

Soap			0.387

Wrong answer	9 (36.0)	11 (44.0)	
Correct answer	16 (64.0)	14 (56.0)	

Onion			0.001

Wrong answer	9 (36.0)	-	
Correct answer	16 (64.0)	25 (100.0)	

*p*-values were calculated using the Fisher's exact test.

Comparing the groups regarding concomitant changes of olfactory and gustatory perception, we found that in the comparison group, normogeusia associated with normosmia was more frequent (10.40%); while in the group of laryngectomized patients there was a higher frequency of hypogeusia and hyposmia (18.72%). In cases where there was involvement of a single perception, hyposmia was more frequent than hypogeusia, especially among the laryngectomized patients (c^²^ = 17.74, *p* < 0.001).

## DISCUSSION

Males, around 60 years of age, represent a worldwide prevalence of laryngeal cancer, as previously reported in the literature^9^, ^10^, ^11^, ^12^ - having been demonstrated in this study - which showed a significant relationship between hyposmia and males among laryngectomized patients, but not hypogeusia.

Research suggests that males have the worst olfactory performance under the hypothesis that genetics and hormonal issues can be determinant for this gender difference in sensory perception^13^. Other researchers point out that the reduction of smell and taste is an occurrence inherent to the physiological process of aging^14^. These variables must also be considered in relation to the sensory losses found in this study, although hyposmia and hypogeusia are reported in the literature as frequent changes in laryngectomized patients^4^, ^15^.

The low level of education found in the laryngectomized group may be associated with socioeconomic and cultural aspects; in addition, the evaluation methods used in our study required a subjective response, taking into account reports or experiences of the subject, which should be considered in understanding the results.

The interpretation of the association between hypogeusia and hyposmia and middle-level education among the laryngectomized subjects deserves caution, because it may be related to socioeconomic status and the type of occupation these patients have.

A study carried out with a standardized test to assess the olfactory function in the Brazilian population found that socioeconomic status may influence the loss of olfactory sensation. Hazardous occupational habits and exposure to pollutants are commonly portrayed in low economic status regions, which may result in olfactory deficit^16^. Another issue is the prevalence of this cancer in rural Brazil^12^, suggesting poorer living conditions and limited access to schooling.

Cigarette smoking and alcohol consumption have been referred to as the main etiological factors for the emergence of such cancer^17^. These harmful practices are also associated with olfactory epithelium degeneration, destruction of taste buds, and neuronal damage, which may result in hyposmia and hipogeusia^18^, ^19^. These findings should be considered, since these habits were significantly more frequent in the laryngectomized participants of this study, and were associated with hypogeusia and hyposmia, it would be interesting to control these variables in subsequent studies.

Regarding the missing teeth, this variable does not seem to have influenced the aspects studied. The similarity of the groups in this item refutes the hypothesis that such variable prevails in laryngectomized patients, although age and educational level may suggest poor oral hygiene, as well as smoking and drinking^20^, and the very treatment of laryngeal cancer may damage the entire oral cavity.

Other researchers^3^, ^6^, ^19^, ^21^ corroborate the hypothesis that the olfactory and taste functions are altered in patients who underwent total laryngectomy - confirmed in our results. Considering the occurrence of hyposmia and hypogeusia significantly greater in those subjects compared to subjects not submitted to laryngectomy, such evidence is still neglected in clinical practice.

The decrease in gustatory function, characterizing hypogeusia, was strongly evident in laryngectomized patients, as well as the occurrence of changes in the distribution of flavors - this features the most important contribution of this study.

Physiologically, flavor perception occurs through taste buds diffusely located on the tongue, palate, epiglottis, pharynx and larynx^22^, ^23^. Sweet and bitter tastes have the same type of intracellular activation through G-protein coupled receptors, while stimulation of salty and sour tastes acts directly on specific ion channels located in the membranes of receptor cells^23^, ^24^, ^25^. However, in all cases, the electrical signals produced by the methods of conversion are uploaded to the central nervous system via cranial nerves VII, IX and X - responsible for the formation of synapses in specific receptor cells^23^, ^26^.

It is believed that the disruption of the complex neurological connections and sensorineural feedback caused by complete removal of the larynx may account for these gustatory changes. It is important to stress that radiation therapy, often associated with the treatment of head and neck cancer, may also result in deleterious effects to sensory organs and tissues, including the oral cavity, tongue, salivary glands, the olfactory epithelium and nerves associated with the perception of smell and taste^27^.

Failure to observe significant changes in the bitter taste in the study groups over the other flavors, may have some explanations. The bitter flavor is part of a body protection mechanism associated with the rejection of certain foodstuffs^28^.

Recent studies have indicated that the bitter taste can be detected at other sites in addition to the tongue and adjacent mouth epithelium, for receptors have been found in the gastric and intestinal mucosae^24^, ^28^, limiting the absorption of bitter tasting dietary toxins that escape aversion in the mouth^28^.

The occurrence of hyposmia in laryngectomized individuals was confirmed in this study, with emphasis in the difficulty of perceiving certain odors. Hyposmia has been evidenced in laryngectomized patients by several authors;^4^, ^6^, ^15^ however, the mechanisms responsible for the decrease in the olfactory function of laryngectomized patients are not yet fully understood. The definitive change in nasal airflow to the tracheostome has been the most cited theory by researchers as the cause of this sensory dysfunction, given that smell depends on chemoreceptors located in the olfactory epithelium to respond to the presence of molecules in the air^4^, ^6^.

Other authors have demonstrated theories related to degenerative diseases of the olfactory epithelium^5^, ^21^ as well as damage caused to the complex sensorineural mechanisms due to the total laryngectomy surgery^29^.

The inability to detect smoke odors and other smell-related signs of danger in laryngectomized patients has been reported in studies, and this may jeopardize the safety of these individuals,^3^, ^19^ similar to the results presented here, in which the identification of “warning odors” was significantly decreased.

The difficulty in the perception of certain odors can influence even food-related pleasures, resulting in weight loss and contributing to malnutrition^19^. Likewise, the loss of the perception of bodily odors may result in socializing difficulties^3^. Our findings suggest that these problems can cause feeding problems and therefore nutritional issues in this population, requiring further investigations in future studies.

Paint thinner - a common solvent in North America - smell interpretation proved unfamiliar to the participants in this study, which may be related to cultural characteristics, and therefore, without significant differences in the results presented here.

Studies detail the association between the sensory functions of smell and taste as responsible for defining the taste of food, closely related to eating habits, nutritional aspects and pleasure during eating^30^, ^31^. It is important to report the significant relationship between the smell and taste functions identified here, with evidence of concomitant hypogeusia and hyposmia in more than half of the total laryngectomized group.

Nasal airflow interruption in the total laryngectomized may not only negatively affect olfactory perception but also their gustatory identification skills, as pointed out in our results. During the olfactory test, we observed facial muscle movements in laryngectomized individuals, suggesting an attempt to force nasal aeration for the consequent stimulation of nasal olfactory epithelium. While not a variable considered in our study, it is important to note that, during chewing, there is the induction of an air stream into the oral cavity by which the olfactory organ is stimulated through the nasopharynx^3^, ^23^. When food approaches the mouth during the respiratory mechanism of inspiration, the olfactory epithelium is also stimulated, thus acting in conjunction with other sensory functions to determine the food flavor^31^, ^32^, ^33^.

The international literature^4^, ^6^, ^21^ has stressed the importance of giving greater emphasis to the treatment of these sensory functions; however, it is limited to only reporting as to the decline of these functions without qualifying with regards to the impaired distribution of tastes and odors.

This study fills this knowledge gap, proving that the perception of salty, sweet and bitter flavors are impaired in laryngectomized individuals, as well as the identification of important warning odors and other smells directly related to feeding, also reduced in this population. Thus, interventions seeking to stimulate the limitations of these sensory functions should be part of the therapeutic scope aimed at individuals submitted to total laryngectomy.

In clinical practice, it is relevant to evaluate these sensory functions and to develop rehabilitation programs for this population, given that the smell and taste alterations may trigger changes in eating habits and impact the pleasure associated with this activity and on the nutritional status of these subjects, in addition to reducing the alert in risky situations, compromising the quality of life of these individuals.

## CONCLUSION

The decrease in gustatory and olfactory functions in laryngectomized individuals was evidenced in this study. In discriminating flavors, the bitter taste was not different between the groups at the expense of other flavors. In the olfactory aspect, the laryngectomized individuals performed worse in detecting warning and food-related odors. It is assumed that the decrease in olfactory function may be directly related to the decrease in the gustatory perception, considering the association between these functions for defining food taste as well as concurrent hypogeusia and hyposmia in laryngectomized individuals.
